# Exogenous carbon monoxide inhibits neutrophil infiltration in LPS-induced sepsis by interfering with FPR1 via p38 MAPK but not GRK2

**DOI:** 10.18632/oncotarget.9084

**Published:** 2016-04-28

**Authors:** Xu Wang, Weiting Qin, Mingming Song, Yisen Zhang, Bingwei Sun

**Affiliations:** ^1^ Department of Burn and Plastic Surgery, Affiliated Hospital, Jiangsu University, Zhenjiang, Jiangsu Province, China

**Keywords:** sepsis, neutrophil, carbon monoxide, microarray genechip, FPR1

## Abstract

Excessive neutrophil infiltration in vital organs is life-threatening to patients who suffer from sepsis. We identified a critical role of exogenous carbon monoxide (CO) in the inhibition of neutrophil infiltration during lipopolysaccharide (LPS)-induced sepsis. CO delivered from carbon monoxide-releasing molecule 2 (CORM-2) dramatically increased the survival rate of C57BL/6 mice subjected to LPS *in vivo*. CORM-2 significantly suppressed neutrophil infiltration in liver and lung as well as markers of inflammatory responses. Affymetrix GeneChip array analysis revealed that the increased expression of chemoattractant receptor formyl peptide receptor 1 (FPR1) may contribute to the excessive neutrophil infiltration. The under agarose migration assay demonstrated that LPS stimulation promoted migration to the ligand of FPR1, N-Formyl-Met-Leu-Phe (fMLP) but that CORM-2 treatment inhibited this promotion. Further studies demonstrated that CORM-2 internalized FPR1 by inhibiting p38 mitogen-activated protein kinase (MAPK) but not G protein-coupled receptor kinase 2 (GRK2), which may explain the inhibitory effect of CORM-2 on LPS-stimulated neutrophils. In summary, our study demonstrates that exogenous CO inhibits sepsis-induced neutrophil infiltration by interfering with FPR1 via p38 MAPK but not GRK2.

## INTRODUCTION

Sepsis is a potentially life-threatening complication of infection with the presence of organ dysfunction [1–3]. Sepsis recently became the leading cause of death in intensive care units (ICUs) [4]. More than 250,000 people die of sepsis annually in the United States. This incidence is rising, despite the increasing input of medical resources [4]. Neutrophils are the first line of innate immune systems that eliminate pathogens in infectious foci [5–7]. However, sepsis-induced immune disorder dramatically contributes to the excessive infiltration of neutrophils in distant organs, multi-organ dysfunction and death [8, 9]. Numerous studies have demonstrated that neutrophil depletion or the suppression of neutrophil migration rescued lethal sepsis [10, 11].

CORMs aim to deliver controlled amounts of CO to tissues and cells. Lipid-soluble metal carbonyl complex tricarbonyl dichlororuthenium(II) dimer ([Ru(CO)3Cl2]2), known as CORM-2, is the first compound to make this technology feasible, and it exhibits broad physiological applicability [12, 13]. Previous studies from our group and others have demonstrated the potential pharmaceutical use of CORM-2 to facilitate sepsis treatment and inhibit the dysfunction of vital organs [14–17]. However, the underlying mechanisms are not known. Whether the therapeutic effect alters excessive neutrophil infiltration and the specific mechanisms are also not known.

The present study used LPS-treated septic mice and mice bone marrow neutrophils as *in vivo* and *in vitro* models, respectively. Affymetrix GeneChip array analysis revealed that FPR1 may be the key molecule that is responsible for excessive neutrophil infiltration. CORM-2 was introduced to explore the effect of exogenous CO on neutrophil infiltration and its potential mechanisms on FPR1-involved neutrophil migration.

## RESULTS

### Effect of CORM-2 on survival and neutrophil infiltration in the liver and lung of septic mice

**Figure 1:**
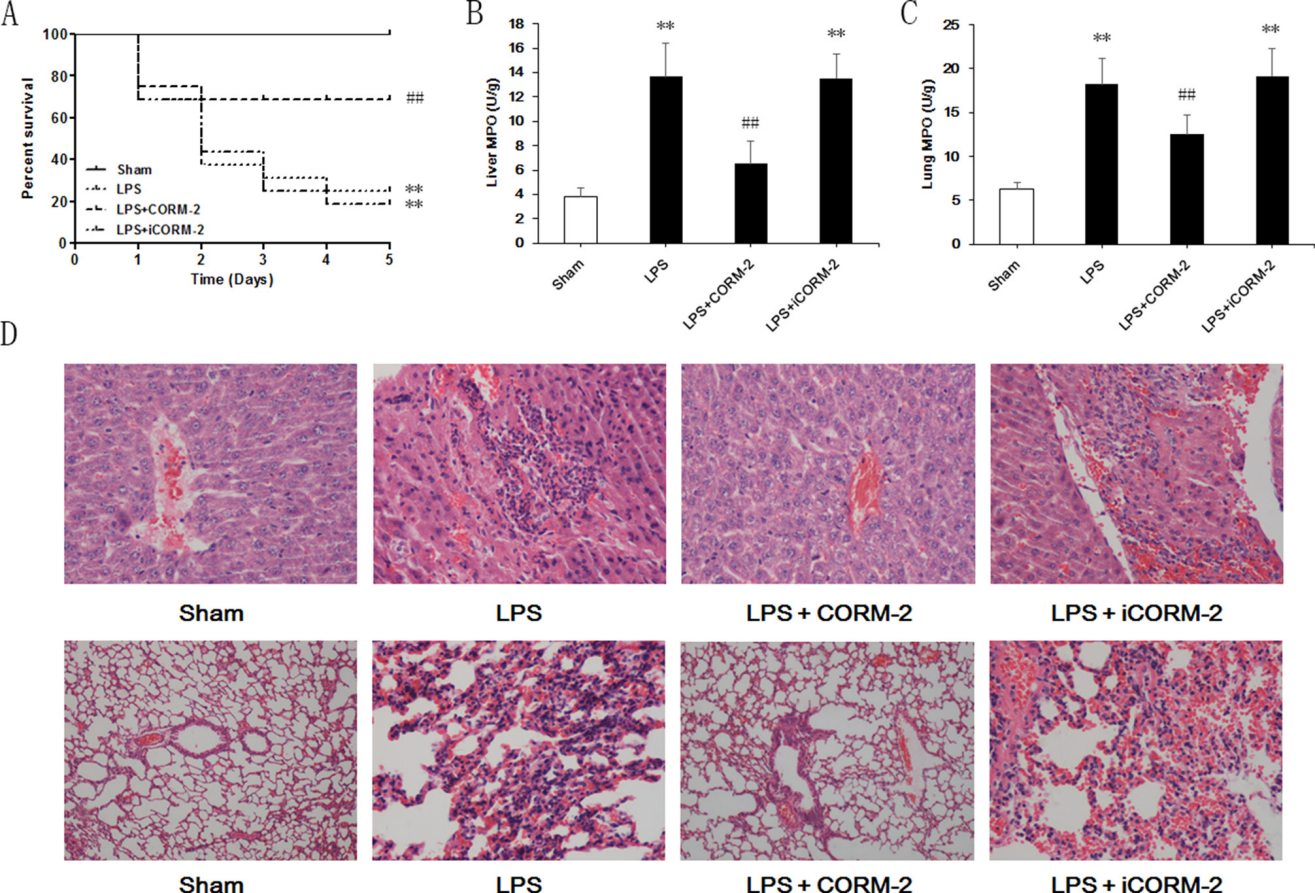
Effect of CORM-2 on survival and neutrophil infiltration in livers and lungs of septic mice C57BL/6 mice received intraperitoneal injections of LPS (15 mg/kg) and CORM-2 or inactive CORM-2 (iCORM-2) was used as the intervention. (**A**) Animal survival was monitored for 5 days after LPS injection. Most of the mice treated with LPS died, and CORM-2, but not iCORM-2, dramatically increased the survival of septic mice from 25% to 68.75%. (**B**, **C**) Liver and lung MPO activity was detected 12 h after LPS injection to evaluate neutrophil infiltration. CORM-2, but not iCORM-2, significantly inhibited the increased MPO activity in livers and lungs after LPS treatment. (**D**) Pathological sections of liver (upper row) and lung (lower row) 12 h after LPS treatment revealed that CORM-2, but not iCORM-2, treatment suppressed tissue injuries and neutrophil infiltration. The data are expressed as the mean ± SD, *n* = 16 for survival analysis and *n* = 10 for other experiment. **P < 0.01 compared to the sham group, ^##^*P* < 0.01 compared to the LPS group.

We performed intraperitoneal LPS injections and applied CORM-2 or iCORM-2 (inactive form of CORM-2) to explore the effect of CORM-2 on lethal sepsis. Sham mice survived for 5 days (Figure 1A). However, the survival rate decreased dramatically 24 h after LPS injection, and only 25% of the mice survived at 5 days. CORM-2-treated septic mice exhibited a significantly increased survival rate of 68.75%. iCORM-2 administration failed to improve the survival of LPS mice. Neutrophil infiltration in liver and lung was evaluated using MPO activity and pathological sections. Liver and lung MPO activity increased significantly in LPS mice compared to sham mice (Figure 1B, 1C). CORM-2, but not iCORM-2, abolished this elevation. Pathological sections of livers in the sham group exhibited complete hepatic lobule structure, normal liver cell morphology and no neutrophil infiltration (Figure 1D). Hepatic cells in the LPS group were swollen, hepatic plates were disarranged, and neutrophil infiltration was visible. CORM-2, but not iCORM-2, intervention alleviated inflammatory changes and neutrophil infiltration. A normal alveolar structure with thin-walled and smooth alveolar septa and no visible infiltration of neutrophils were observed in the lung sections of the sham group. The LPS group exhibited diffuse pulmonary edema, pulmonary capillary expansion, thickening of the alveolar septa, visible oozing and red blood cell extravasation in the alveolar space. Neutrophil infiltration into the interstitium was apparent. CORM-2, but not iCORM-2, exerted inhibitory effects on tissue injury and neutrophil infiltration.

### Effect of CORM-2 on inflammatory responses in livers and lungs of septic mice

**Figure 2:**
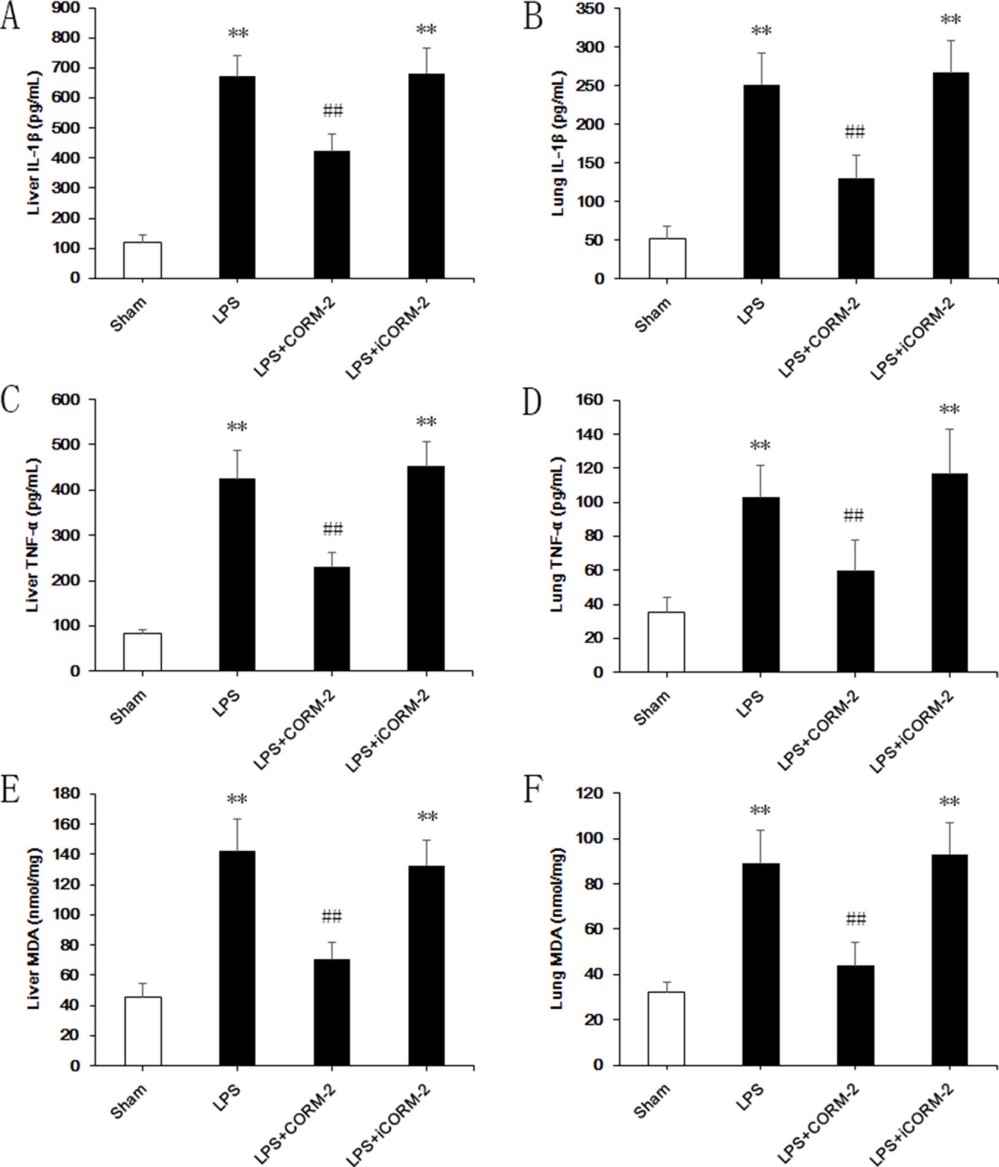
Effect of CORM-2 on inflammatory responses of livers and lungs in septic mice C57BL/6 mice received intraperitoneal injections of LPS (15 mg/kg), and CORM-2 or inactive CORM-2 (iCORM-2) was used as the intervention. Mice were euthanized 12 h after LPS treatment, and liver and lung tissues were harvested for assessments. (**A**, **B**) LPS markedly increased the expression of interleukin 1β (IL-1β) in liver and lung. CORM-2, but not iCORM-2, administration abolished this elevation. (**C**, **D**) Elevation of tumor necrosis factor α (TNF-α) was also inhibited by CORM-2 administration in liver and lung after LPS treatment. (**E**, **F**) Elevation of malondialdehyde (MDA) content was inhibited by CORM-2 administration in liver and lung after LPS treatment. The data are expressed as the mean ± SD, *n* = 10 for each group. **P < 0.01 compared to the sham group, ^##^*P* < 0.01 compared to the LPS group.

Low expression of pro-inflammatory cytokines IL-1β (Figure 2A, 2B) and TNF-α (Figure 2C, 2D) in sham mice were measured in liver and lung homogenates. LPS treatment dramatically increased the expression of IL-1β (liver, 671.5 ± 69.1 vs. 120.6 ± 24.1; lung, 251.8 ± 40.3 vs. 51.9 ± 17.2 pg/mL) and TNF-α (liver, 424.4 ± 64.3 vs. 82.5 ± 10.1; lung, 102.8 ± 18.6 vs. 35.5 ± 8.2 pg/mL) compared to the sham group. However, significant reductions of IL-1β (liver, 422.3 ± 60 vs. 671.5 ± 69.1; lung, 130 ± 30.5 vs. 251.8 ± 40.3 pg/mL) and TNF-α (liver, 229.3 ± 32.7 vs. 424.4 ± 64.3; lung, 59.6 ± 18 vs. 102.8 ± 18.6 pg/mL) were achieved following CORM-2 administration in LPS mice. No amelioration was observed in the iCORM-2 group. Elevation of MDA, as an indicator of oxidative stress, (liver, 70.4 ± 11.7 vs. 142 ± 21.3, lung, 43.8 ± 10.2 vs. 89 ± 14.7 nmol/mg) was also significantly abolished by CORM-2 but not iCORM-2 treatment (Figure 2E, 2F).

### Effect of LPS stimulation on the expression of neutrophil chemoattractant receptors

**Figure 3:**
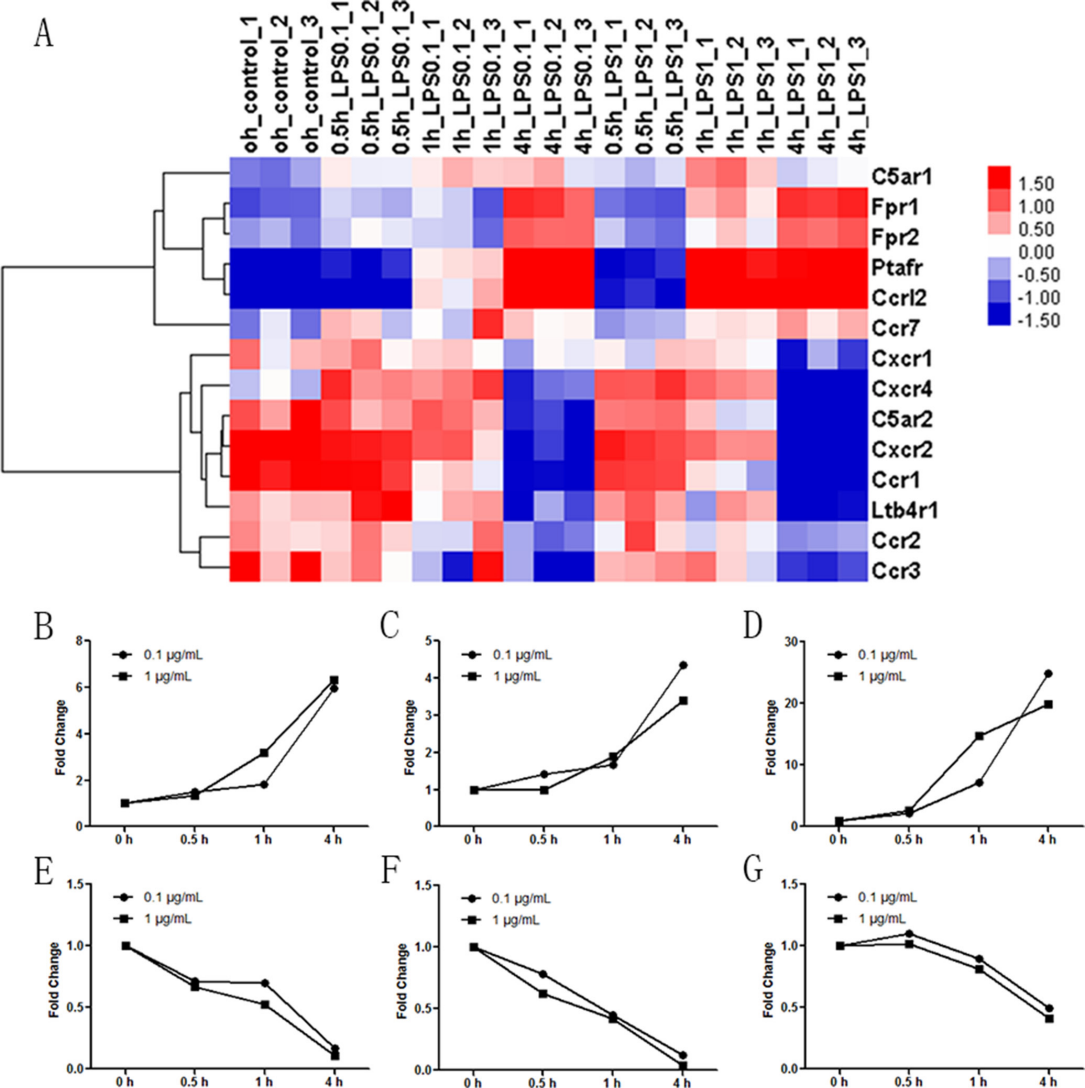
Effect of LPS stimulation on the expression of neutrophil chemoattractant receptors Neutrophils were stimulated with 0.1 μg/mL or 1 μg/mL LPS for 0.5 h, 1 h, or 4 h. Untreated neutrophils were used as control. (**A**) Cluster analyses of Affymetrix GeneChip array revealed that receptors for fMLP (FPR1 and FPR2), PAF (PTAFR) and CC receptor-like 2 (CCRL2) were markedly increased after 0.1 μg/mL and 1 μg/mL LPS stimulation for 0.5 h, 1 h and 4 h. Expression of the C5a receptor (C5aR) 1 was slightly increased after stimulation with 0.1 μg/mL LPS but decreased after stimulation with 1 μg/mL LPS for 4 h. Expression of CCR7 was slightly increased by 1 μg/mL but not 0.1 μg/mL LPS stimulation. The expression of C5aR2, CXC chemokine receptor (CXCR) 2, CXCR4, CC chemokine receptor (CCR) 1, CCR2 and CCR3 decreased after 0.1 μg/mL and 1 μg/mL LPS stimulation for 0.5 h, 1 h and 4 h. CXCR1 expression was not altered after 0.1 μg/mL LPS stimulation, but it decreased after 1 μg/mL LPS stimulation. LTB4 receptor (LTB4R1) expression was not altered after 1 μg/mL LPS stimulation, but it decreased after 0.1 μg/mL LPS stimulation. (**B**–**G**) FPR1, FPR2, PTAFR, C5aR2, CXCR2 and CCR2 were selected for RT-PCR assay to validate the array data. Other chemoattractant receptors including CCR4, CCR5, CCR6, CCR8, CCR9, CXCR2, CXCR3, CXCR5, CXCR6 and CXCR7 were not different between groups, and these data are not shown. *n* = 3 for genechip and *n* = 5 for RT-PCR.

Cluster analyses of the Affymetrix GeneChip array investigated the effect of LPS stimulation on the expression of neutrophil chemoattractant receptors (Figure 3A). The expression of complement 5a receptor 1 (C5aR1), FPR1, FPR2, platelet-activating factor receptor (PTAFR), and CC receptor-like 2 (CCRL2) increased in LPS-stimulated neutrophils. The expressions of CXC chemokine receptor 1 (CXCR1), CXCR4, C5aR2, CXCR2, CC chemokine receptor 1 (CCR1), leukotriene B4 receptor (LTB4R1), CCR2 and CCR3 decreased in LPS-stimulated neutrophils. Three significantly increased mRNAs (FPR1, FPR2 and PTAFR, Figure 3B–3D) and three significantly decreased mRNAs (C5aR2, CXCR2 and CCR2, Figure 3E–3G) were selected for RT-PCR assay to validate the array data. The results demonstrated that FPR1 was 5.97-fold higher 4 h after LPS 0.1 μg/mL and 6.35-fold higher 4 h after LPS 1 μg/mL compared to the control group. The increases of FPR2 were less significant, with fold changes of 4.37 and 3.41, respectively. Fold increases for 4 h PTAFR expression were 24.77 and 19.81 in the presence of 0.1 and 1 μg/mL LPS, respectively. C5aR2 expression decreased. The results revealed 0.17- and 0.11-fold decreases after 4 h of stimulation with 0.1 and 1 μg/mL LPS, respectively. CXCR2 expression decreased 0.12- and 0.04-fold in the presence of 0.1 and 1 μg/mL LPS, respectively. The fold changes of CCR2 were 0.49 and 0.41 4 h after stimulation of 0.1 and 1 μg/mL LPS, respectively. Other chemoattractant receptors, including CCR4, CCR5, CCR6, CCR8, CCR9, CXCR2, CXCR3, CXCR5, CXCR6 and CXCR7, were not different between groups, and these data are not shown. Overall, these data confirmed that FPR1, FPR2 and PTAFR were the three significantly increased neutrophil functional chemotaxis receptors. FPR2 mediates FPR1 desensitization, and it did not cause neutrophil migration [18]. PTAFR may account for the impairment of neutrophil migration [19]. Future studies will investigate the role of FPR1 in neutrophil migration.

### Effect of CORM-2 on the migration of LPS-stimulated neutrophils

**Figure 4:**
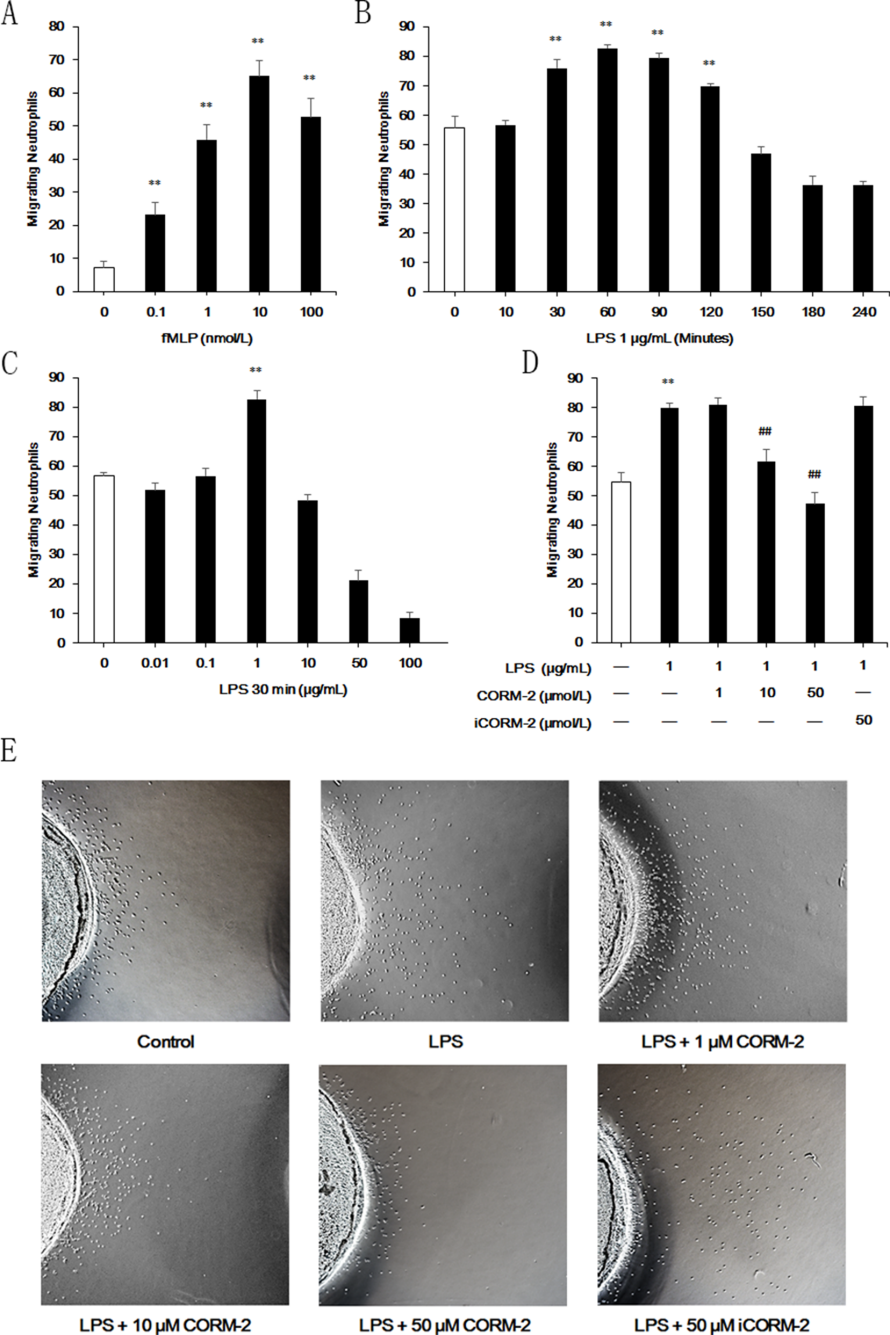
Effect of CORM-2 on migration in LPS-stimulated neutrophils Under agarose migration assays were used to examine the ability of neutrophils to migrate to fMLP. (**A**) 10 nmol/L fMLP induced more neutrophil migration than other doses. (**B**) Stimulation with 1 μg/mL LPS improved neutrophil migration to fMLP from 30 min to 120 min. (**C**) Stimulation with LPS at 1 μg/mL for 30 min promoted neutrophil migration. (**D**) Enhancement of neutrophil migration after LPS stimulation (1 μg/mL, 30 min) was suppressed by CORM-2 in a dose-dependent manner. (**E**) Representative images of under agarose neutrophil migration. The data are expressed as the mean ± SD, *n* = 5 for each group. **P < 0.01 compared to the control group, ^##^*P* < 0.01 compared to the LPS group.

The under agarose migration assays were performed to investigate neutrophil migration, and fMLP was used as a chemoattractant. The results demonstrated that 10 nmol/L fMLP attracted the most neutrophils (Figure 4A), and this concentration was thus used in subsequent experiments. Neutrophils were stimulated with LPS for the indicated times and doses. The results demonstrated that LPS treatment (1 μg/mL) for 30 min to 120 min significantly enhanced neutrophil migration (Figure 4B). LPS stimulation failed to increase neutrophil migration from 150 min to 240 min compared with the control groups at the indicated times, but LPS promoted neutrophil migration (data not shown). We found a significant increase in migrating neutrophils 30 min after LPS stimulation only when neutrophils were stimulated with 1 μg/mL LPS (Figure 4C). Therefore, stimulation with 1 μg/mL LPS for 30 min was used in subsequent experiments. Cells were incubated with LPS and 1 μmol/L, 10 μmol/L, 50 μmol/L CORM-2 or 50 μmol/L iCORM-2 to investigate the effect of CORM-2 on LPS-stimulated neutrophils migrating to fMLP (Figure 4D). Migrating neutrophil numbers were not altered when co-incubated with 1 μmol/L CORM-2, but 10 μmol/L CORM-2 significantly inhibited LPS-induced neutrophil migration, and 50 μmol/L CORM-2 exerted a better effect. Representative images of under agarose neutrophil migration are shown in Figure 4E.

### Effect of CORM-2 on apoptosis, phagocytosis and TLR4 expression in LPS-stimulated neutrophils

**Figure 5:**
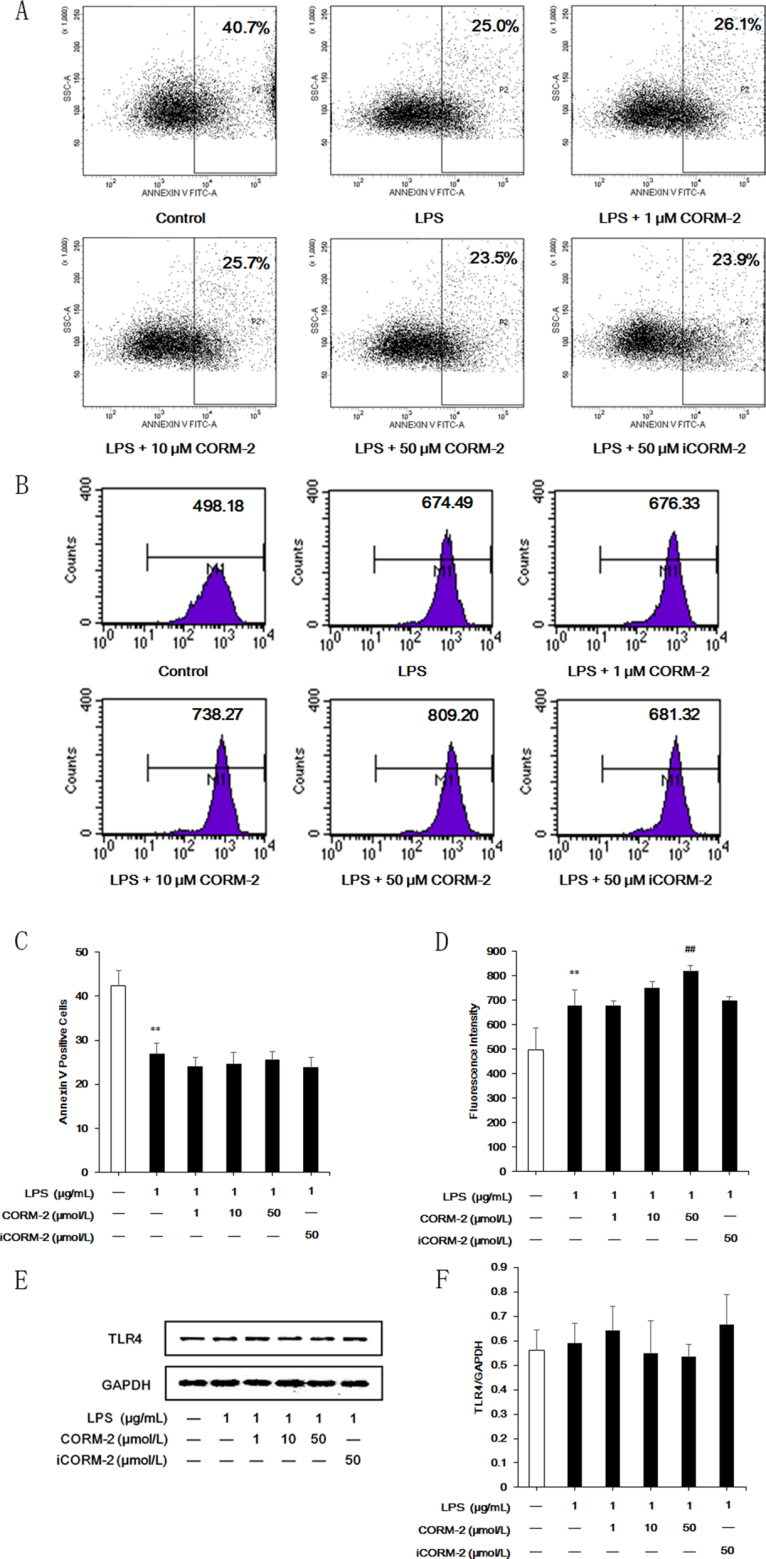
Effect of CORM-2 on apoptosis, phagocytosis and TLR4 expression in LPS-stimulated neutrophils (**A**) Decreased apoptotic rate was observed following LPS stimulation, but no significant differences in apoptotic rate were discovered after CORM-2 treatment. (**B**) LPS stimulation enhanced neutrophil phagocytic activity. Intervention with 50 μmol/L CORM-2 significantly reinforced the LPS-induced increased phagocytic activity. (**C**, **D**) Statistical analyses of neutrophil apoptosis and phagocytosis. (**E**) Western blot results show that TLR4 expression was not affected by CORM-2. (**F**) Statistical analyses of western blot results. The data are expressed as the mean ± SD, *n* = 5 for each group. **P < 0.01 compared to the control group, ^##^*P* < 0.01 compared to the LPS group.

Differences in the apoptotic rates were detected using Annexin V staining and FCM to exclude the possibility that the inhibitory effect of CORM-2 was the result of increased apoptosis. LPS stimulation suppressed neutrophil apoptosis, which is consistent with previous reports. CORM-2 did not affect neutrophil apoptosis (Figure 5A, 5C). LPS stimulation enhanced neutrophil phagocytic activity, and CORM-2 intervention reinforced this effect (Figure 5B, 5D). CORM-2 intervention did not alter the expression of the receptor for LPS, TLR4 (Figure 5E, 5F).

### Effect of CORM-2 on FPR1 internalization and GRK2 and p38 MAPK activation in LPS-stimulated neutrophils

**Figure 6:**
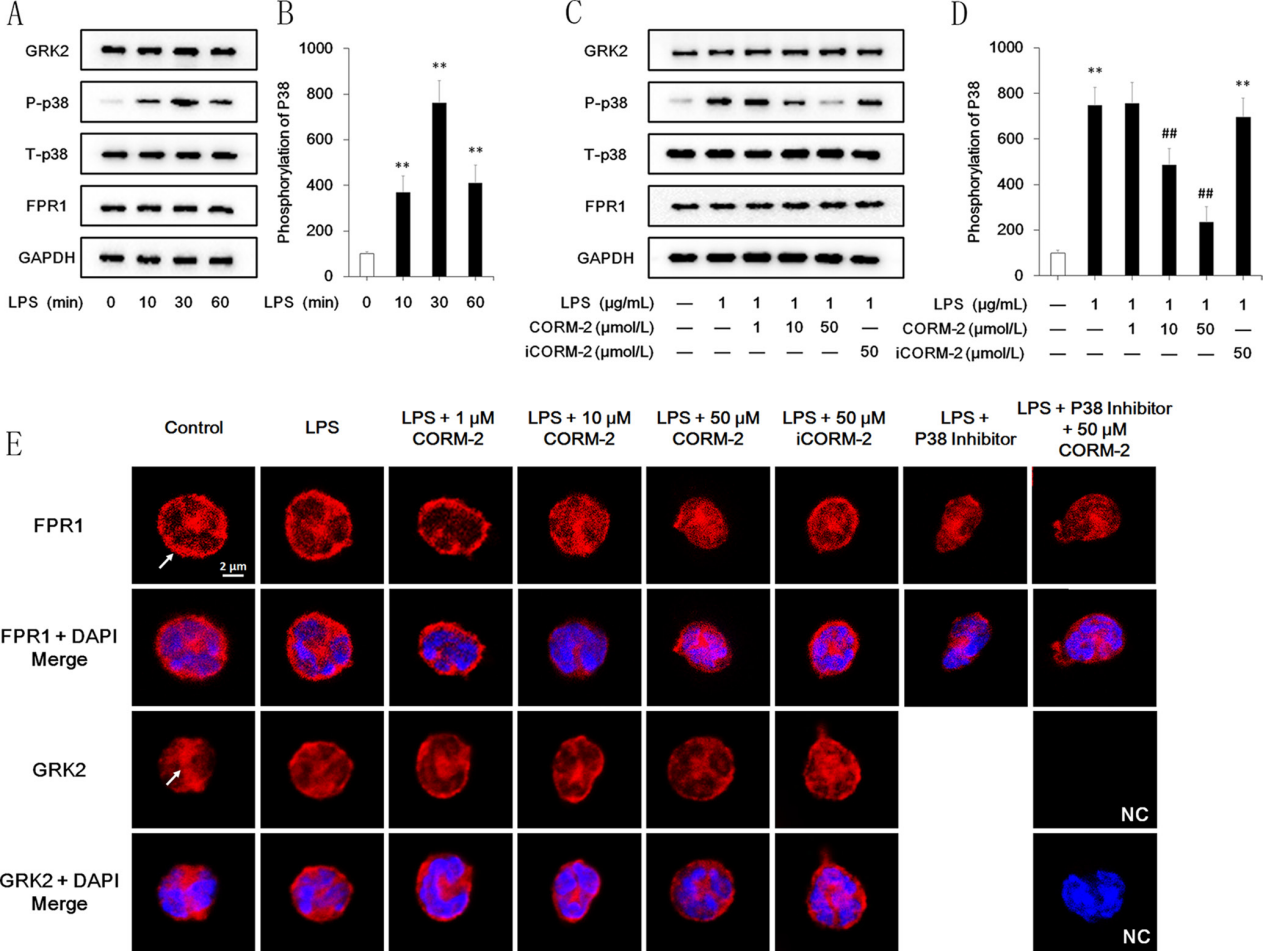
Effect of CORM-2 on FPR1, GRK2 and p38 MAPK in LPS-stimulated neutrophils (**A**) LPS stimulation significantly increased p38 MPAK phosphorylation at 10 min, 30 min and 60 min compared to the control group. LPS stimulation did not alter GRK2 and FPR1 expression. (**B**) Quantified analyses revealed that LPS dramatically increased the phosphorylation of p38 MAPK. No significant differences in GRK2 and FPR1 were observed, and the quantified results are not displayed. (**C**) Administration of CORM-2 inhibited p38 MAPK phosphorylation but not the expression of GRK2 or FPR1 in a dose-dependent manner. (**D**) Quantified analyses revealed that CORM-2 inhibited p38 MAPK phosphorylation in a dose-dependent manner. (**E**) LPS (1 μg/mL, 30 min)-stimulated neutrophils were treated with CORM-2 or iCORM-2 at the indicated doses. Confocal microscopy images revealed that CORM-2 but not iCORM-2 internalized FPR1 (arrow) in LPS-stimulated neutrophils, and a p38 inhibitor exerted a similar effect. Translocation of GRK2 (arrow) to the cellular membrane after LPS stimulation was observed, which indicated an improved function of GRK2. Neither CORM-2 nor iCORM-2 suppressed the translocation of GRK2. The negative control (NC) showed that non-specific signals were not detected. The data are expressed as the mean ± SD, *n* = 5 for each group. **P < 0.01 compared to the control group, ^##^*P* < 0.01 compared to the LPS group.

Quantification of western blot images revealed that the FPR1 expression levels were not altered after LPS stimulation for 10 min, 30 min, or 60 min, and representative images are shown in Figure 6A. FPR1 protein expression was slightly, but not significantly, increased 240 min after LPS stimulation (data not shown). CORM-2 intervention did not decrease the expression level of FPR1 protein (Figure 6C) but induced internalization of FPR1. Confocal microscopy revealed that 50 μmol/L CORM-2 obtained the best effect (Figure 6E). Treatment with a p38 inhibitor alone or in combination with CORM-2 exerted similar effects. GRK2 activation after LPS stimulation was evidenced by membrane translocation (Figure 6E). However, CORM-2 intervention failed to reverse GRK2 translocation. The western blotting demonstrated significant p38 MAPK phosphorylation (Figure 6A, 6B) after LPS stimulation at 10 min, 30 min and 60 min, but GRK2 expression was not altered (Figure 6A). CORM-2 intervention inhibited p38 MAPK phosphorylation, and 50 μmol/L CORM-2 was the optimal concentration, which parallels the results of the under agarose migration assay (Figure 6C, 6D).

### Effect of p38 MAPK and GRK2 on LPS-stimulated neutrophil migration

**Figure 7:**
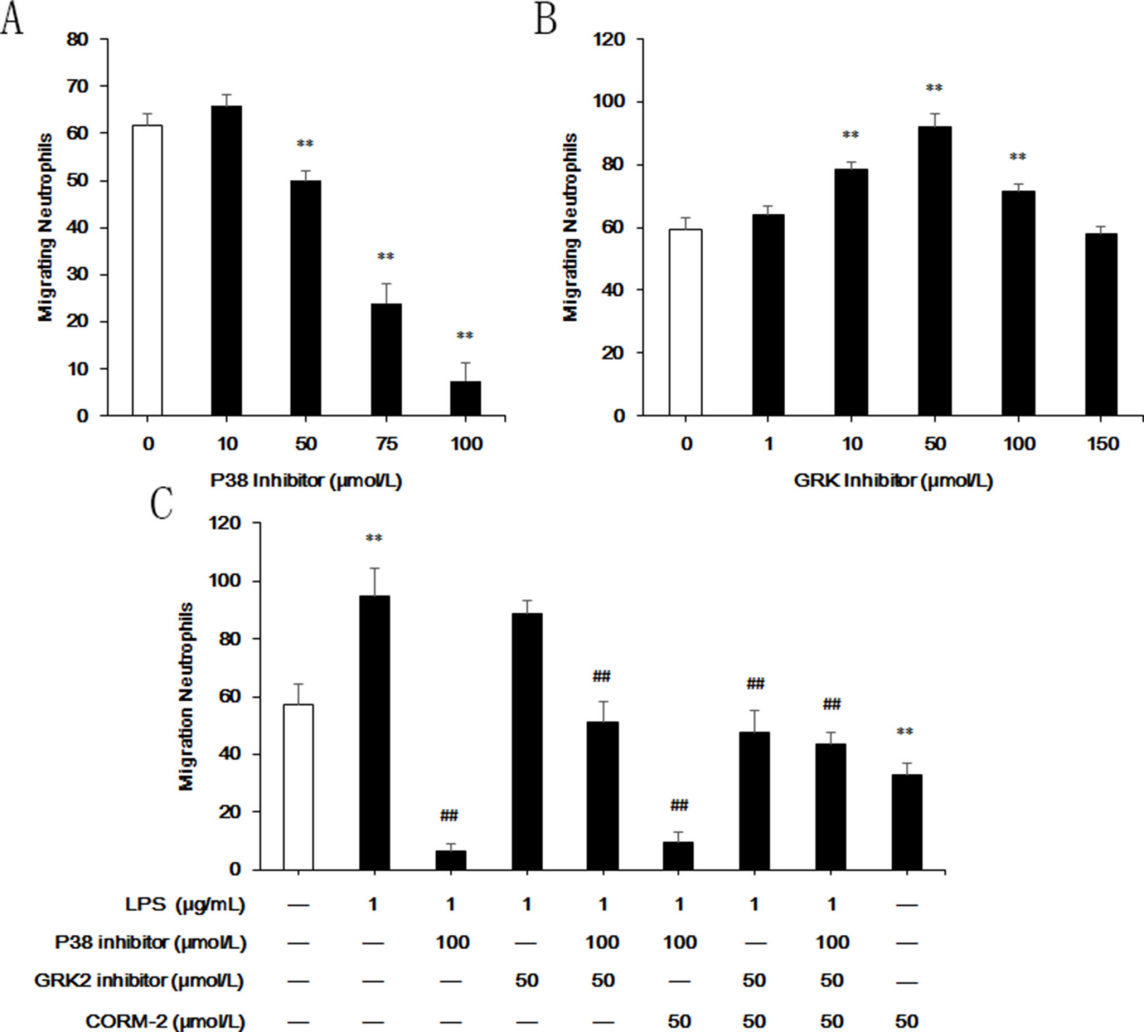
Effect of p38 MAPK and GRK2 on LPS-stimulated neutrophil migration Under agarose migration assays were used to investigate the ability of neutrophils to migrate to fMLP. (**A**) Pre-incubation with SB203580 for 30 min was used to inhibit p38 MAPK. Neutrophil migration was dramatically inhibited, and 100 μmol/L SB203580 almost abolished neutrophil migration to fMLP. (**B**) Pre-incubation with 4-amino-5-(bromomethyl)-2-methylpyrimidine hydrobromide for 30 min was used to inhibit GRK2. A concentration of 50 μmol/L of the GRK inhibitor optimally promoted neutrophil migration. (**C**) Neither a single stimulation of LPS nor co-intervention with CORM-2 increased the number of migrating neutrophils when pre-incubated with a p38 inhibitor. Pre-treatment with a GRK inhibitor failed to increase LPS-stimulated migrating neutrophils, but it restored the migration of p38 inhibitor pre-treated neutrophils. CORM-2 treatment abolished the increase in migrating neutrophils in the LPS + GRK inhibitor group but not the LPS + p38 inhibitor + GRK inhibitor group. CORM-2 suppressed neutrophil migration in the absence of LPS. **P < 0.01 compared to the control group, ^##^*P* < 0.01 compared to the LPS group.

P38 MAPK is a noncanonical GRK that is indispensable for neutrophil migration to fMLP via phosphorylation of its receptor, FPR1, which blocks FPR1 GRK2-induced internalization. The present study used SB203580 as a p38 MAPK inhibitor and 4-amino-5-(bromomethyl)-2-methylpyrimidine hydrobromide as a nonselective GRK inhibitor. Under agarose migration assays demonstrated that the p38 MAPK inhibitor prevented neutrophil migration and that the GRK inhibitor promoted migration (Figure 7A, 7B). The optimal concentration of the p38 MAPK inhibitor and GRK inhibitor were 100 μmol/L and 50 μmol/L, respectively, and these concentrations were used in subsequent experiments. LPS stimulation did not induce neutrophil migration when pre-incubated with the p38 inhibitor (Figure 7C). Treatment with LPS + GRK inhibitor did not increase migration compared to the LPS group, but this treatment restored the migration of p38 inhibitor pre-treated neutrophils. CORM-2 exerted no effect on the LPS + p38 inhibitor, the LPS + p38 inhibitor group, or the LPS + GRK inhibitor group, but it abolished the increase of migrating neutrophils in the LPS + GRK inhibitor group. CORM-2 suppressed neutrophil migration in the absence of LPS.

## DISCUSSION

Sepsis is a severe, urgent medical condition that substantially affects the lives of affected patients [20, 21]. Recent studies have demonstrated that severe infection-induced disorders of the immune system is the predominate cause of septic death [22–24]. Neutrophil function is the first line of immune defenses to eliminate pathogens at the early stage of sepsis, but these cells are paralyzed with sepsis progression [25, 26]. The directionality of neutrophils is significantly harmed in sepsis. The failure to recruit neutrophils to infectious foci and excessive infiltration in distant organs account for spread of the infection and vital organ dysfunction [26, 27]. Previous studies demonstrated that neutrophil depletion and the suppression of neutrophil migration dramatically inhibit the excessive infiltration of neutrophils and improve various biomarkers of sepsis [10, 11].

Endogenous CO is a byproduct generated by heme oxygenase 1 (HO-1) that is a critical signaling molecule in numerous vital cellular events [28–30]. Previous studies from our group and others have demonstrated the beneficial effect of CORM-2-delivered exogenous CO in lethal sepsis [14–17]. However, the underlying mechanisms are not known because of the complicated physiological nature of CO. LPS-induced sepsis is deadly for experimental mice [31]. Only 25% of septic mice survived after 5 days of observation. Notably, CORM-2, but not iCORM-2, dramatically increased the survival rate to 68.75%. Neutrophil infiltration into vital organs, such as liver and lung, was assayed as a principal contributor to sepsis mortality. MPO activity and pathological images were used as indictors of neutrophil infiltration [32], and the results demonstrated that CORM-2 significantly suppressed the elevation of neutrophil numbers in tissues. Sepsis induced serious inflammatory pathological injuries and increased potent pro-inflammatory cytokines TNF-α and IL-1β, which were suppressed by CORM-2, but not iCORM-2, intervention. CORM-2 also decreased the MDA levels, which is an indicator of lipid peroxidation, in liver and lung of LPS mice. This beneficial effect may be due to the inhibition of neutrophil infiltration [33, 34] and the overall cell redox-modifying potential of CORM-2 [35–37].

The mechanisms of accumulated neutrophils in vital organs are not definite because of the complexity and acute nature of sepsis. Chemoattractant receptors for neutrophil infiltration are highly important, and the Affymetrix GeneChip array analysis was used to identify potential targets. LPS is the sepsis-associated pro-inflammatory stimulus derived from the outer membrane of Gram-negative bacteria, and it was used to mimic the inflammatory response *in vitro* [27]. Cluster analyses of Genechip arrays revealed that the expression of FPR1, FPR2 and PTAFR increased markedly, and this increase was corroborated using RT-PCR. FPR2 likely mediates the desensitization of FPR1, and it did not cause neutrophil migration [18]. PTAFR may underlie the impairment of neutrophil migration [19]. Future studies will focus on the role of FPR1 in neutrophil migration.

The efficacies of CO on neutrophils during acute inflammation and sepsis were demonstrated in various animal models. However, whether CO is involved in chemoattractant receptor FPR1-mediated neutrophil migration is not known. Under agarose migration assays were performed, and the potent chemotactic ligand to FPR1 (fMLP) was used as a chemoattractant to further determine whether LPS promoted FPR1-involved neutrophil migration and the inhibitory effect of CORM-2. The results demonstrated that LPS stimulation dramatically promoted neutrophil migration to fMLP in time- and dose-dependent manners. The effect of CORM-2 on neutrophil migration was evaluated. Neutrophils were stimulated with LPS and treated with the indicated doses of CORM-2 or iCORM-2. The results demonstrated that increasing CORM-2 doses inhibited fMLP-induced neutrophil migration. However, our results are inconsistent with earlier reports, which suggested that CO enhanced human neutrophil random migration [38] and activation [39]. Differences in species, CO donors and migration models may contribute to these discrepancies. Migration function may be affected by PMN apoptotic status. Therefore, we evaluated the apoptotic rate in each group to exclude the possibility that this inhibitory effect was the result of apoptosis. The results demonstrated that LPS stimulation inhibited the apoptotic rate, which is consistent with previous studies [40], and that CORM-2 failed to alter the apoptotic rate in LPS-stimulated neutrophils. The unchanged PMN apoptotic status indicated that the suppressive effect of CORM-2 on PMN migration is not related to PMN apoptosis. CORM-2 intervention in LPS-stimulated neutrophils reinforced phagocytic activity. Different signaling pathways mediate PMN migration and phagocytic activity. Migration and phagocytosis are somewhat inconsistent because phagocytosis decreases the expression of chemokine receptors in cellular membranes and inhibits neutrophil migration [41, 42]. CORM-2 intervention did not alter the expression of the LPS receptor TLR4, which suggests that the effect of CORM-2 on neutrophil function was independent of TLR4 expression.

Genechip analyses demonstrated that mRNA expression of FPR1 increased significantly when neutrophils were stimulated with 1 μg/mL LPS for 1 h and 4 h. However, western blotting revealed that the expression level of FPR1 protein was not altered. FPR1 protein expression was slightly, but not significantly, increased in neutrophils that were stimulated for 4 h. The incompatible expression between mRNA and protein revealed the post-transcriptional or post-translational effects of LPS. However, the unaltered expression of FPR1 is critical for the perseverance of the functional response to fMLP, compared with the decreased expression of CXCR1 and CXCR2 and the impaired response to its ligand, IL-8, in murine sepsis [43]. The cellular location of FPR1 is also critical for neutrophil chemotaxis [44].

**Figure 8:**
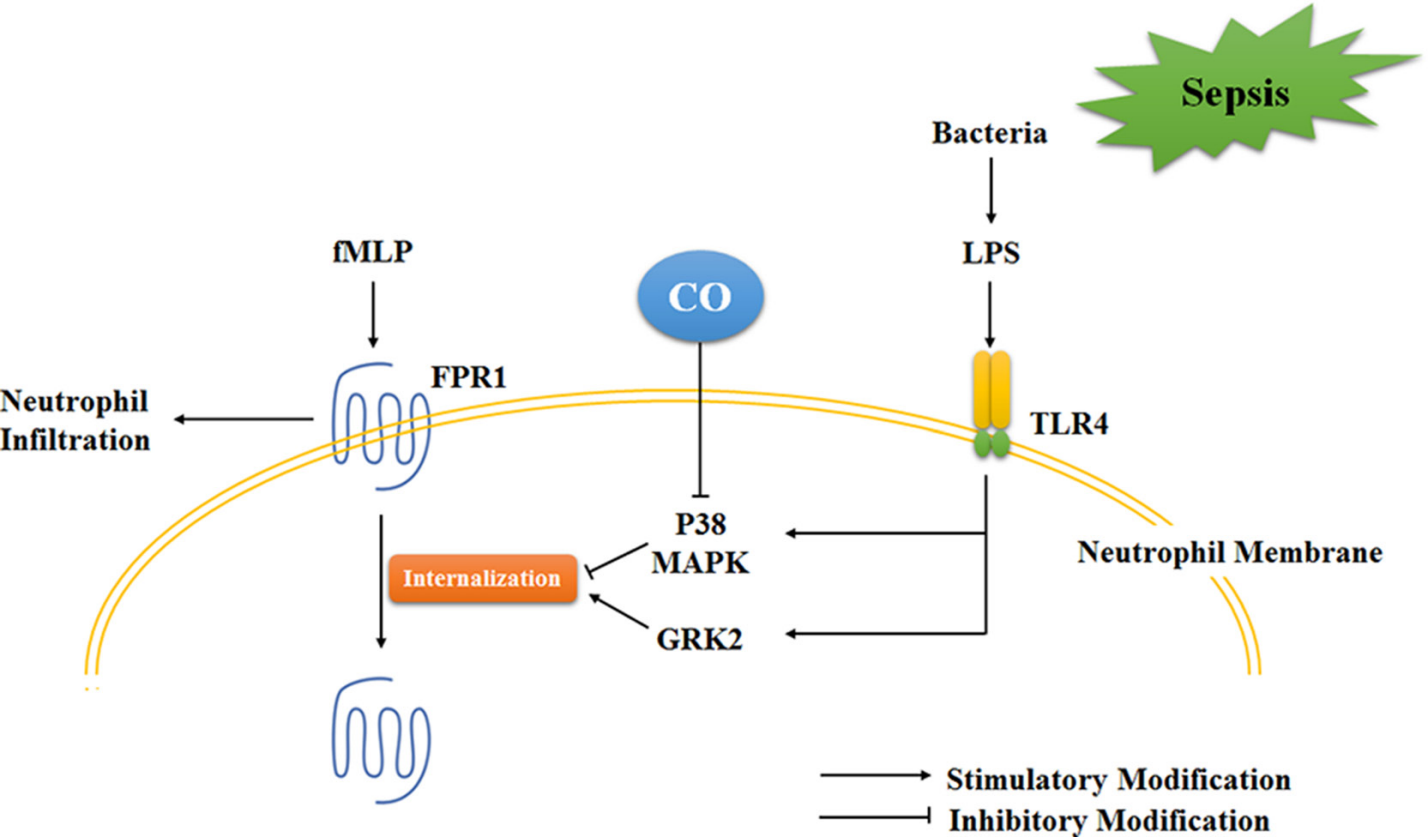
Schematic illustration of the mechanism of CO inhibition of neutrophil migration during sepsis The present data suggest that LPS stimulation activated p38 phosphorylation and GRK2 translocation, but only p38 phosphorylation was inhibited by CO intervention. Therefore, CO inhibited LPS-stimulated FPR1-involved neutrophil infiltration.

We evaluated the expression and distribution of FPR1 to examine the inhibitory mechanisms of CORM-2 on FPR1-involved neutrophil migration. The results demonstrated that CORM-2 significantly promoted the internalization of FPR1 to the cellular membrane instead of FPR1 expression in LPS-stimulated neutrophils, which indicated that the internalization of FPR1 may be the inhibitory cause of CORM-2. Notably, p38 MAPK and GRK2 are two critical molecules that regulate FPR1 internalization by exerting opposite effects [45, 46]. GRK2 acts as an inhibitor of FPR1 via FPR1 phosphorylation and internalization, and phosphorylated p38 MAPK is an activator of FPR1 by phosphorylating FPR1, which blocks GRK2 from recognizing and internalizing. LPS stimulation did not alter the expression level of GRK2 and p38 MAPK based on the results of genechip and western blots. But LPS translocated GRK2 to the cellular membrane, which was not affected by CORM-2 intervention. In contrast, LPS stimulation activated p38 MAPK phosphorylation, and CORM-2 significantly suppressed this phosphorylation. The inhibition of p38 alone or in combination with CORM-2 internalized FPR1. These data suggest that LPS stimulation activated p38 phosphorylation and GRK2 translocation, but only p38 phosphorylation was inhibited by CORM-2 intervention (Figure 8). Notably, CO exerted discriminatory effects on p38 phosphorylation depending on the different stimuli and cell types because of its complex bioactivities. CO inhibits p38 phosphorylation in cytomix-stimulated Caco-2 cells [47] and TNF-α-stimulated EC [48], but it promotes p38 phosphorylation in LPS-stimulated macrophages [49] and H9C2 cells [50].

We used a p38 MAPK inhibitor and GRK inhibitor to further verify this mechanism. Under agarose chemotaxis assays demonstrated that a p38 MAPK inhibitor abolished neutrophil migration to fMLP, but a GRK inhibitor enhanced neutrophil migration. The p38 inhibitor abolished neutrophil migration in LPS-stimulated cells, but the GRK inhibitor failed to increase neutrophil migration, which might be caused by the limited number of migrating cells in the under agarose system [51] because the migration of neutrophils to fMLP with the GRK inhibitor was significantly restored in p38 inhibitor pre-treated neutrophils. CORM-2 inhibited neutrophil migration in the LPS + GRK inhibitor group but failed to inhibit neutrophil migration in the LPS + GRK inhibitor + p38 inhibitor group because of the inhibitory effect on p38 phosphorylation. CORM-2 suppressed p38-dependent neutrophil migration in the absence of LPS stimulation because of the inhibitory effect on p38 phosphorylation.

The *in vivo* and *in vitro* data support a therapeutic role of CORM-2 in sepsis by interfering with excessive neutrophil infiltration in liver and lung via a p38 MAPK, but not GRK2, pathway. LPS is generally not detected in the plasma of septic patients, and there are obvious differences in species, age, the presence of co-morbidities and the timing of treatment between the endotoxemia model and human sepsis. Animal studies are essential to provide the necessary framework and understand the therapeutic targets in a relatively concise condition. However, there is a large gap in the application of CORM-2 as a treatment for clinical sepsis. CO gas has already passed safety evaluation in Phase I testing in healthy human beings, as a type of transitional metal carbonyl, but CORM-2 must be stringently characterized from a metabolic and toxicological standpoint.

## MATERIALS AND METHODS

### Materials

CORM-2, DMSO, RIPA and LPS were purchased from Sigma-Aldrich (MO, USA). CORM-2 was solubilized in DMSO to obtain a 40 mmol/L stock. An inactive form of CORM-2 (negative controls) was used in some experiments and prepared as follows: iCORM-2 was inactivated form of CORM-2 by leaving the stock of CORM-2 at 37°C in a 5% CO_2_ humidified atmosphere for 24 h to liberate CO. The iCORM-2 solution was bubbled with nitrogen to remove the residual CO present in the solution. 1 × or 10 × HBSS with or without Ca^2+^ Mg^2+^, RMPI 1640 medium, fetal bovine serum (FBS) and agarose were purchased from Life Technologies (CA, USA). Antibodies to p38 mitogen-activated protein kinase (MAPK) and p-p38 MAPK and the p38 MAPK inhibitor SB203580 were purchased from Cell Signaling Technology (MA, USA). Antibodies to FPR1 and G protein-coupled receptor kinase 2 (GRK2) and the GRK inhibitor 4-amino-5-(bromomethyl)-2-methylpyrimidine hydrobromide were purchased from Santa Cruz Biotechnology (TX, USA). All other chemicals were of reagent grade and obtained from Sigma unless otherwise stated.

### Treatment of mice

C57BL/6 mice (body weight 20 ± 2 g, Experimental Animal Center of Jiangsu University, Zhenjiang, Jiangsu, China) were given free access to a normal mouse diet and tap water. All mice were randomly assigned to 4 groups: Sham group (*n* = 10); LPS group (*n* = 10); LPS + CORM-2 group (*n* = 10); and LPS + inactive CORM-2 (iCORM-2) group (*n* = 10). Mice in the sham group received intraperitoneal injections of normal saline, and mice in the LPS groups received intraperitoneal injections of LPS (15 mg/kg). Mice in the LPS + CORM-2 and LPS + iCORM-2 groups received the same treatment with the immediate administration of CORM-2 (8 mg/kg, i.v.) or iCORM-2 (8 mg/kg, i.v.), respectively. Mice were euthanized 12 h after LPS injection, and liver and lung tissues were harvested.

### Isolation and preparation of mouse bone marrow neutrophils

Isolation of mouse bone marrow neutrophils was performed according to previous protocols [52]. Mice were euthanized, and femurs and tibias were removed. The ends of the bones were resected, and the bone marrow in each bone was harvested into 50-mL centrifuge tubes through a 70 μm cell strainer via perfusion of 3 mL of an ice-cold neutrophil isolation buffer (HBSS with 0.1% bovine serum albumin without Ca^2+^ or Mg^2+^). Marrow cells were pelleted in a centrifuge (600 x *g*, 4°C, 5 minutes) and resuspended in 2 mL of a neutrophil isolation buffer. The cell solution was placed over a discontinuous Percoll gradient consisting of a stock Percoll solution (90% Percoll, 10% 10 × HBSS without Ca^2+^ or Mg^2+^) diluted to 78%, 69% and 52% in HBSS. The cell solution was spun at 1500 g at 4°C for 30 minutes. Purified murine neutrophils localized to a band between the 78% and 69% layers. This band was collected with a transfer pipette, washed in neutrophil isolation buffer and suspended in HBSS with Ca^2+^ and Mg^2+^ + 1% FBS at 1.0 × 10^7^ cells/mL. Purity was greater than 97% as assayed using flow cytometry and an FITC-labeled anti-Ly- 6G antibody (Cell Signaling Technology, Massachusetts, USA).

The indicated doses and times of LPS administration were used to stimulate neutrophils with or without the presence of CORM-2 or iCORM-2. The p38 MAPK inhibitor and GRK inhibitor were pre-incubated with neutrophils for 30 min before the above-listed interventions. The neutrophils were washed twice with ice-cold PBS and added to the wells.

### Survival

C57BL/6 male mice were randomly assigned to 4 groups: Sham group (*n* = 16), LPS group (*n* = 16), LPS + CORM-2 group (*n* = 16), and LPS + iCORM-2 group (*n* = 16). All mice had normal access for water and food and monitored for 5 days.

### Assessment of neutrophil infiltration in lung and liver

Samples of 10% formalin fixed lung tissue were embedded in paraffin and sectioned at 5 μm for routine histology. Slides were stained with hematoxylin and eosin, and an experienced pathologist evaluated the slides in a blinded manner. Inflammatory organ injuries and neutrophil infiltration were assessed. Myeloperoxidase (MPO) activity (Nanjing Jiancheng, Jiangsu, China) was detected according to the manufacturer's instructions.

### Evaluation of inflammatory responses in lung and liver

Homogenates of lung and liver tissue were performed using a mechanical homogenate. Malondialdehyde (MDA) (Nanjing Jiancheng, Jiangsu, China), interleukin 1β (IL-1β) and tumor necrosis factor α (TNF-α) (Qiaoyi, Shanghai, China) were detected according to the manufacturer's instructions.

### Genechip array analysis

Neutrophils were stimulated with 0.1 μg/mL or 1 μg/mL LPS for 0.5 h, 1 h or 4 h and an unstimulated group (0 h) was used as control. Briefly, total RNA was isolated using a QIAGEN RNeasy Mini Kit. Total RNA (500 ng) was converted to synthesize double-stranded complementary DNA (cDNA), and double-stranded cDNA was labeled and hybridized to an Affymetrix Mouse Transcriptome Assay 1.0 genechip. Slides were hybridized and washed, and processed slides were scanned using an Affymetrix GeneChip2 Scanner 3000 7G. The data were analyzed using Affymetrix Genechip software, and significantly altered expression of chemoattractant receptors are shown.

### RT-PCR

Three increased mRNAs (FPR1, FPR2 and PTAFR) and three decreased mRNAs (C5aR2, CXCR2 and CCR2) were selected for RT-PCR to validate the array data. First-strand complimentary DNA (cDNA) was synthesized using the RevertAid First Strand cDNA Synthesis Kit (Thermo, MA, USA). Real-time qPCR was performed using Maxima SYBR Green/ROX qPCR Master Mix (Thermo, Waltham, MA, USA). Relative quantitative levels of samples were calculated using the 2^−ΔΔCT^ method, and the results are expressed as a fold-change by normalizing the expression of the target genes to a housekeeping gene (GAPDH). Table 1 presents the primers.

**Table 1:** Sequences of the used primers

Gene	Forward primer	Reverse primer
FPR1	ACAGCCTGTACTTTCGAC	CTGGAAGTTAGAGCCCGTTC
FPR2	GTCAAGATCAACAGAAGAAACC	GGGCTCTCTCAAGACTATAAGG
PTAFR	TCGATACACGCTCTTTCCGA	GTCAGCCATAGTGAGATTCACCATA
C5aR2	ATGGCCGACTTGCTTTGT	CCTTGGTCACCGCACTTTC
CXCR2	GGTCGTACTGCGTATCCTGCCTCA	TAGCCATGATCTTGAGAAGTCCAT
CCR2	TGGTAAATTCTTCAGCTTTTCC	TCCACAACCTGATAAAGCCTCC
GAPDH	CACCCCATTTGATGTTAGTG	CCATTTGCAGTGGCAAAG

### Under agarose migration assay

The under agarose migration assay was performed as previously described [51]. Falcon Petri dishes (35 mm) were filled with 3 mL of a 1.2% agarose solution containing 50% HBSS with Ca^2+^ and Mg^2+^ and 50% RPMI 1640 culture medium containing 20% heat-inactivated FBS. A straight line was cut into the gel of 2 wells, 3.5 mm in diameter and 2.4 mm apart, after the agarose solidified. The gels were equilibrated for 1 h in a 37°C/5% CO_2_ incubator. fMLP (10 μL) at the indicated doses was added to the left well, and 10 μL of neutrophils were added to the right well. Gels were incubated for 3 h in a 37°C /5% CO_2_ incubator. The results were observed at 100 × magnification using a microscope, and the absolute number of migrating neutrophils was calculated.

### Quantitation of apoptosis

Neutrophils from each group were collected and washed twice with cold PBS. Annexin V was added to the cells and gently vortexed. Annexin V (Vazyme Biotech, Jiangsu, China) is a Ca^2+^-dependent phospholipid-binding protein with high affinity for PS, and it binds to exposed apoptotic cell surface PS. Cells were incubated for 15 min at room temperature in the dark and analyzed using FC within 1 h.

### Phagocytosis assay

Phagocytic activity of neutrophils was detected using the pHrodo *E. coli* Bioparticles Phagocytosis kit (Life Technologies, CA, USA). Neutrophils from each group were collected and washed twice with cold PBS. Neutrophils were mixed with pHrodo *E. coli* and incubated for 1 h at 37°C. The engulfed bacteria displayed fluorescence when in the low pH environment of the acidified phagocytic compartment. Phagocytosis was assayed using FC within 1 h.

### Western blot

Neutrophils from each group were washed with ice cold PBS for two times. RIPA buffer that contained protease and phosphatase inhibitor cocktails was added for lysis of cells. The lysates were incubated with 3 × SDS buffer, boiled and loaded on 10% SDS–PAGE gels. 20 μg of protein were subjected to electrophoresis on 10% SDS polyacrylamide gels, with the use of the discontinuous system and transferred onto nitrocellulose membranes. The membranes were incubated with primary antibody and followed by secondary antibody conjugated to horseradish peroxidase (3:5000). ECL reagent was used to visualize bands with FluorChem FC3 (ProteinSimple, USA) and AlphaView 3.4.0 software was used for quantified analysis. GRK2, total p38 (T-p38) and FPR1 was normalized to GAPDH and phosphorylated p38 (P-p38) was normalized to T-p38. Percent of control was presented.

### Confocal laser-scanning microscope

Neutrophil suspensions from each group were transferred to micro centrifuges and washed with ice cold PBS for two times. Then neutrophils were fixed with 4% paraformaldehyde and permeabilized with 0.3% Triton X-100. 2% BSA in 10% goat serum was used for blocking and 1:200 anti FPR1 antibody was incubated overnight. 1:500 secondary antibody was incubated for 1 h. DAPI was stained for 10 min. Distributions of FPR1 and GRK2 were observed using the Leica SP8 confocal laser-scanning microscope (Leica, Germany). Negative control (NC) was performed under the same conditions but primary antibody was not incubated.

### Statistical analysis

GraphPad Prism 5 was used for the statistical analysis of all data. Values are presented as the mean ± SD. One-way factorial analysis of variance (ANOVA) and Tukey's test for the comparisons were performed. Survival was analyzed using the log-rank test. *P* < 0.05 was considered statistically significant.

## Abbreviations

CORM-2: Carbon monoxide releasing molecule 2
FPR1: formyl peptide receptor 1
DMSO: dimethyl sulfoxide
MAPK: mitogen-activated protein kinase
GRK2: G protein-coupled receptor kinase 2
fMLP: N-Formyl-Met-Leu-Phe
ANOVA: One-way factorial analysis of variance
LTB4: Leukotriene B4
PAF: platelet activating factor.
